# In Vivo Fluorine Imaging Using 1.5 Tesla MRI for Depiction of Experimental Myocarditis in a Rodent Animal Model

**DOI:** 10.1155/2023/4659041

**Published:** 2023-07-14

**Authors:** Thore Dietrich, Stephan Theodor Bujak, Thorsten Keller, Bernhard Schnackenburg, Riad Bourayou, Rolf Gebker, Kristof Graf, Eckart Fleck

**Affiliations:** ^1^Department of Cardiology, Deutsches Herzzentrum Berlin, Berlin 13353, Germany; ^2^Department of Geriatrics, Krankenhaus Hedwigshöhe, Alexianer St. Hedwig Kliniken Berlin GmbH, Berlin 12526, Germany; ^3^Charité-Universitätsmedizin Berlin, Corporate Member of Freie Universität Berlin and Humboldt-Universität zu Berlin, Berlin 10117, Germany; ^4^B. Braun Melsungen AG, Melsungen 34212, Germany; ^5^Philips Healthcare, Hamburg 22335, Germany; ^6^Department of Cardiology, Jüdisches Krankenhaus Berlin, Berlin 13347, Germany

## Abstract

The usefulness of perfluorocarbon nanoemulsions for the imaging of experimental myocarditis has been demonstrated in a high-field 9.4 Tesla MRI scanner. Our proof-of-concept study investigated the imaging capacity of PFC-based ^19^F/^1^H MRI in an animal myocarditis model using a clinical field strength of 1.5 Tesla. To induce experimental myocarditis, five male rats (weight ~300 g, age ~50 days) were treated with one application per week of doxorubicin (2 mg/kg BW) over a period of six weeks. Three control animals received the identical volume of sodium chloride 0.9% instead. Following week six, all animals received a single 4 ml injection of an 20% oil-in-water perfluorooctylbromide nanoemulsion 24 hours prior to *in vivo*^1^H/^19^F imaging on a 1.5 Tesla MRI. After euthanasia, cardiac histology and immunohistochemistry using CD68/ED1 macrophage antibodies were performed, measuring the inflamed myocardium in *μ*m^2^ for further statistical analysis to compare the extent of the inflammation with the ^19^F-MRI signal intensity. All animals treated with doxorubicin showed a specific signal in the myocardium, while no myocardial signal could be detected in the control group. Additionally, the doxorubicin group showed a significantly higher SNR for ^19^F and a stronger CD68/ED1 immunhistoreactivity compared to the control group. This proof-of-concept study demonstrates that perfluorocarbon nanoemulsions could be detected in an *in vivo* experimental myocarditis model at a currently clinically relevant field strength.

## 1. Introduction

For noninvasive diagnostic strategies, cardiac magnetic resonance (CMR) is a widely accepted imaging modality used to detect the presence of myocardial inflammation [1]. As described in the Lake Louise criteria using the late gadolinium enhancement (LGE) protocol, CMR can detect tissue oedema as well as irreversible myocardial damage (e.g., necrosis, fibrosis) [2, 3]. However, these characteristics lack specificity to discriminate inflammation from other pathologies, such as oedema and scar tissue [4, 5]. And despite having compelling imaging sensitivity, MRI only provides indirect signs of cardiac inflammation. Fluorine imaging has two decisive advantages: (1)the natural concentration of fluorine in the body is extremely low, thus allowing for a vanishingly low background signal and (2) the high sensitivity of the ^19^F kernel, reaching 83% of the hydrogen's, is a favourable prerequisite for the obtention of a signal [6, 7]. The use of perfluorocarbon (PFC) nanoemulsions has the significant advantage to provide us with the possibility to image directly the inflammation process. The format of an oil-in-water nanoemulsion proves advantageous: once in the bloodstream, the nanodroplets containing the PFCs are phagocytosed by circulating monocytes and macrophages that are migrating towards the inflammatory site. This provides the mechanism of “natural” enrichment in fluorine atoms at the inflammation foci that is the underlying principle of this imaging strategy. PFCs have been shown to be biologically inert, and they are completely excreted within days to weeks through exhalation [8].

The potential of *in vivo* inflammation imaging using PFC-laden nanoparticles and ^19^F MRI has been demonstrated in experimental cardiac and cerebral ischemia [9], tumor [10], pneumonia [11], as well as myocardial infarction [12]. However, the application for *in vivo* diagnosis of experimental myocarditis was so far restrained to the ultrahigh field strength of 9.4 Tesla [13, 14]. Thus, the aim of the present study was to evaluate the utility of a PFC-laden nanoemulsion for the detection of myocarditis using a rodent model down to 1.5 Tesla MRI while also relying on protocols established in clinical practice.

## 2. Materials and Methods

### 2.1. Myocarditis Animal Model

**Figure 1:**
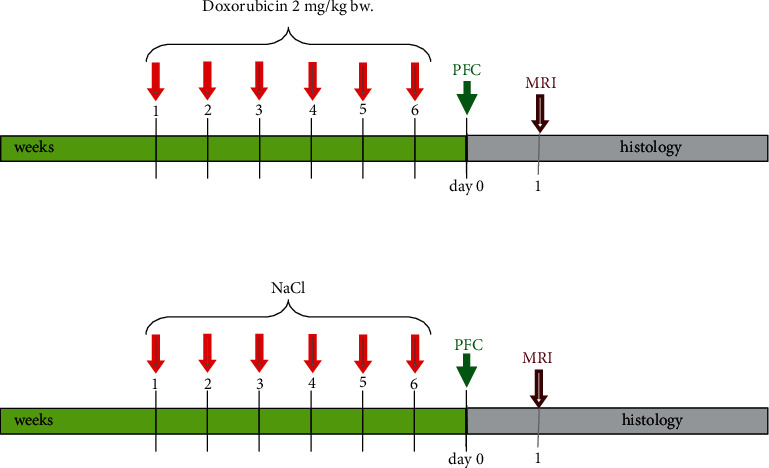
Experimental design used in this study. Animals from the doxorubicin group (upper part of the graphics) or from the control group (lower part) received 6 weekly injections before receiving a single (4 mL) injection of PFC-laden nanoparticles prior to MR-imaging. The animals were then euthanized, and their myocardium was investigated by means of histology and immunohistochemistry.

Eight ~300 g male CD (SD)-rats (Charles River, Wilmington, USA) at an age of about 50 days were used in this study. Five animals received an i.v. administration of 2 mg/kg/bw of doxorubicin (Hexal, Holzkirchen, Germany) through the tail vein once a week under general isoflurane inhaled anesthesia applied via a nose cone (1.5–2 vol% with 1 L oxygen flow) over a period of six weeks. The same protocol was applied to the control group consisting of three animals, save for the injection of doxorubicin, which had been replaced by a volume-matched injection of NaCl solution. One week after the end of the (doxorubicin or saline) treatment, all animals received a single injection of 4 mL PFC nanoemulsion and were then examined 24 hours later using ^1^H and ^19^F MRI, also under isoflurane inhaled anesthesia applied via a nose cone (1.5–2 vol% with 1 L oxygen flow).  Figure 1 demonstrates the experimental design of this study.

All experiments were performed under local legislation and with the permission of the federal institutions under the approval number G0180/10 and have given care according to the Guide for the Care and Use of Laboratory Animals (http://www.nap.edu).

### 2.2. Composition of the Perfluoroctylbromid (PFOB) Nanoemulsion

The PFC nanoemulsion tested in this study is an oil-in-water emulsion consisting of distilled water consisting of 20% *w*/*v* perfluorooctylbromide (ABCR GmbH, Karlsruhe, Germany) as the oil phase, 2% *w*/*v* lipoid E80 as an emulsifier, and 2.5% glycerol as an isotonic agent. For the production of the nanoemulsion, glycerol and lipoid E80 are initially dispersed in distilled water using an ultra Turrax homogenizer (IKA Labortechnik GmbH & Co.KG, Staufen, Germany). Then perfluoroctylbromid (PFOB) was added under continuous dispersion, and the resulting preemulsion was homogenized three times at 750 bar with a laboratory table-top high pressure homogenizer Panda Plus 2000 (GEA Niro Soavi, Lübeck, Germany). The average particle size of 160 nm and polydispersion index of 0.17 of the nanoemulsion were inferred by means of dynamic light scattering analysis (Nicomp 380 DLS, Anasysta, USA).

### 2.3. ^1^H and ^19^F- Multinuclei Imaging at 1.5 Tesla Clinical Scanners

The MRI system used in this experiment was a 1.5 T Philips Achieva (Philips, Best, Netherlands) with a Nova dual gradient system (amplitude 33 mT/m, max. Slew rate 180 mT/m/ms). The scanner was equipped with the hardware multinuclei package to allow for ^19^F imaging. The acquisition was performed using dual-tuned ^1^H/^19^F transmit/receive coil (RAPID Biomedical, Rimpar, Germany), consisting of a solenoid coil of 70 mm inner diameter, oriented perpendicularly to the field B_0_. Imaging using ^1^H and ^19^F occurred in sequence: the protocol consisted of a 3D Turbo-Spin-Echo anatomical (^1^H) scan followed by an ultrashort TE balanced steady-state free precession sequence (UTE-bSSFP) for fluorine imaging. The UTE-bSSFP is a spin-density-weighted sequence that has superior sensitivity and is provided with a 3D radial readout [15]. The short echo time, significantly shorter than achievable with standard gradient echo sequences, allows for efficient detection of the signal before magnetization loss through relaxation has yet fully set in. Furthermore, the use of UTE-bSSFP allowed us to tackle two specific problems in the spectral domain. First, many spectral lines of PFOB can be made to fit in the bandwidth of one voxel, reducing blurring [16]. The second problem is the cancellation of the signal from these lines due to their different rotation frequencies, and it is avoided through measurement before the dephasing effects become too strong [15]. We hence decided to proceed empirically and explored a large parameter space by varying TE, TR, and the flip-angle using in-vitro samples containing diluted concentrations of the nanoemulsion of perfluorooctylbromide (PFOB). The flip angle used in the final acquisition sequence maximizes the available signal intensity and desired resolution of 3 mm; the value of 20° was found to be optimal.

The acquisition was performed with a 3D radial readout (FOV 120 mm, isotropic voxel 3 mm^3^, flip angle *α* = 20°, excitation bandwidth of 5 kHz centred on the PFOB-CF_2_ line group, pixel bandwidth of 1084 Hz, TR/TE of 2.8 ms/120 *μ*s, 32 signal averages, scan duration 11 min). The acquisition on animal subjects was repeated at least once to ensure the reproducibility of the detected fluorine signal. The anatomical T2-weighted Turbo Spin Echo 3D sequence TR/TE = 2000/95 ms, scan duration 6 minutes) imaged the very same tissue volume at the same resolution. Proton imaging provided us with the anatomical locus of the detected fluorine enrichment. For the evaluation of the signal-to-noise ratio of the ^19^F signals, the DICOM datasets were loaded into the software ImageJ (National Institute of Health, USA). For each animal in both groups, we set regions of interest (ROI) in the relevant cardiac slices enclosing the whole myocardium to determine the ^19^F signal. To calculate the noise strength, we used the anatomical scan (^1^H) to place a ROI outside the animal, transposed this ROI onto the ^19^F image of the same slice, and determined the noise strength as the standard deviation of the ^19^F signal in this area. When the enhancement signal in the myocardium was detected over many slices, this procedure for the determination of the signa-to-noise ratio was repeated for all of them; the highest value was retained and subsequently used as input in the statistical analysis.

### 2.4. Imaging Data Processing and SNR Determination

During our study, we realized that it was necessary to develop a convenient way to superimpose the ^19^F and the ^1^H in our routine if we wanted to streamline the visual recognition of fluorine enrichment. The MRI acquisition 3D datasets were exported in the PAR/REC Philips proprietary format for further processing with in-house software developed in MATLAB (Mathworks, Natick/MA, USA). Noise statistics and optional noise thresholding were performed on the fluorine data to create maximum intensity projections (MIP) of the ^1^H and ^19^F datasets together. We introduced color coding for better discrimination of the fluorine enrichment signals (12-bit “jet” colormap) from the anatomical features (8-bit grey colormap).

**Figure 2:**
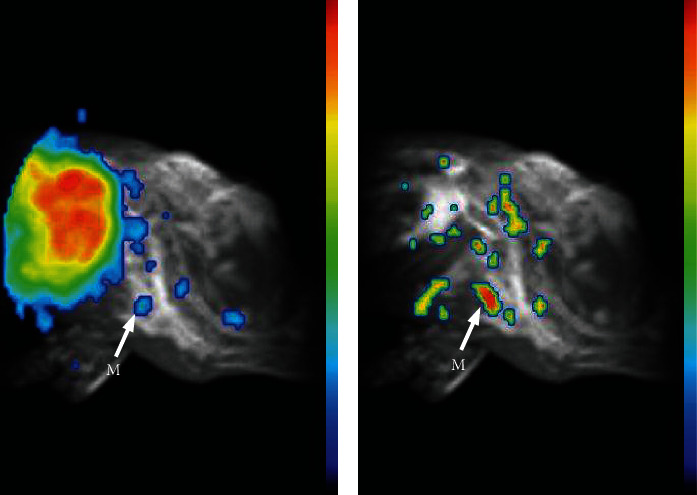
A MIP of the overlaid ^19^F signal over the anatomical scan in 1.5 Tesla MRI: (a) The fluorine in the liver is predominant on the image, and small signals in other organs are merely visible. Through cluster analysis, unspecific signals can be identified and subsequently excluded from the visualization by recomputing the colormap, leading to (b). The visual identification of weaker ^19^F enhancements (the marker M stands for the signal in the myocardium in this example) is thus facilitated.

Due to the capability of ^19^F nanoemulsions to enrich in inflammation as well as in macrophages of the reticuloendothelial system (RES) [17], we detected a high signal from the liver. This high signal would act like a “tree that hides the forest” phenomenon as it stretches the colormap, and smaller signals like the myocardial inflammation in this study cannot be displayed efficiently. While there exists at least one technique to reduce extensive signals from the liver [18], it was not used in this study. As a result, the liver signal clearly dominates all other signals in the ^1^H/^19^F overlays using colormap. Alterations of the colormap (for instance, linear to log) brought little success in enhancing the readability of the overlays. We settled for a more computer-intensive solution using in-house-developed software where the signal in the liver could be recognized in the 3D dataset. This technique relied on finding the liver signal blob using a 3D-cluster analysis of the ^19^F dataset. The colormap is then recalculated using the min-max values of the remaining (and less intense) clusters. In the ^19^F/^1^H overlaid MRI images presented in this publication, the ^19^F signal is devoid of the liver signal; we chose not to display it along the rest of the ^19^F signal to facilitate the discovery, localization, and comparison of the fainter signals. An example is given in Figure 2.

We are aware that focusing on weak signals at the expense of bigger ones in the ^1^H/^19^F overlays is the main limitation of this approach. However, this cluster-driven image processing is absolutely independent of the mathematical evaluation of the SNR, which has been performed using the raw ^19^F dataset using ImageJ.

### 2.5. Histology and Immunohistochemistry

After euthanasia, hearts were removed and snap-frozen at -80°C. Axial cryosections were cut to a thickness of 7 *μ*m for subsequent immunohistochemistry. Every second cut was stained with haematoxylin-eosin (H.E.). Inflammatory cells were detected by specific antibodies using the labelled streptavidin-biotin method. Briefly, anti-CD68/ED1 (dilution 1 : 50; Santa Cruz Biotechnology Inc., Santa Cruz, USA) was used to detect macrophages as primary antibody. A goat anti-mouse IgG antibody (dilution 1 : 500; Jackson Immunoresearch Laboratories Inc., West Grove, USA) was used as a secondary antibody. Images were acquired with an Olympus BX61 microscope equipped with a CCD-camera (ColorView II, Olympus Germany). Quantification of CD68/ED1 positive cells was performed using Cell-F software (Olympus, Hamburg, Germany), measuring positively stained areas in *μ*m^2^ using color threshold values [19, 20].

### 2.6. Statistical Analyses for the Immunochemistry

All statistical analyses were done with SPSS Statistics 20.0 (IBM, Armonk, NY). To conform to the small populations used in this study, the groups of doxorubicin and control rats were compared with the Wilcoxon-Mann–Whitney test. The significance level was set to a *p* value <0.05. Values are given as the mean ± standard deviation. The correlation was tested with the Spearman rank test and two-sided significance.

## 3. Results

### 3.1. MRI-Scans

**Figure 3:**
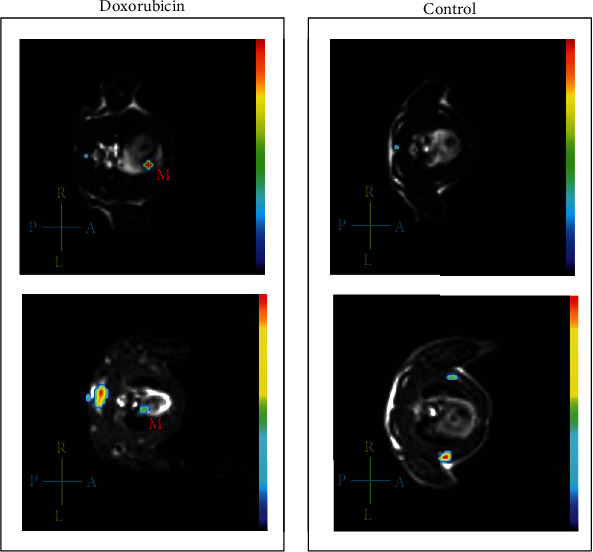
^19^F signal (color overlay) over the ^1^H anatomical scan in 1.5 Tesla MRI (black and white). The red label “M” marks the myocardial fluorine signal. All other displayed signals are attributed to enhancement in the lymph nodes, especially in the dorsal thoracic region, where a large collection of lymph nodes near the shoulder blades is located [21].

A clear ^19^F MRI enhancement could be detected in all animals. 24 hours after injection of the contrast agent, no signal was detectable in the vessels, and all animals exhibited a signal in the liver and spleen (not shown). We associated these signals with the temporary sequestration of the PFC nanoparticles in cells of the reticuloendothelial system. Sparse signals, attributable to the lymph nodes, were also present. However, a robust signal in the heart was only detectable in animals in the doxorubicin-treated group. An illustration is provided in Figure 3, where the signals measured in two animals of each group are displayed. We can distinguish the signals from the myocardium (label “M” on the two left images, doxorubicin group animals), while the other significant hot spots in all images, irrespective of the group, are associated with lymph nodes in the axillary and spine regions [21].

**Figure 4:**
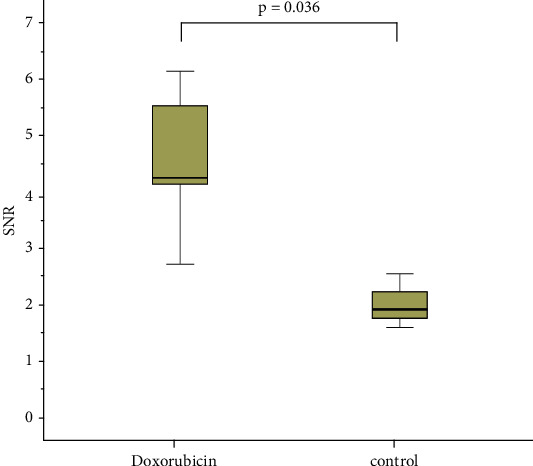
Statistical analysis of the SNR in a myocardial ROI. The signal of the doxorubicin group was significantly higher compared to the control group (4.56 ± 1.19 vs. 2.03 ± 0.39; *p* = 0.036).

In the statistics analysis, the SNR, measured by using a myocardial ROI of the doxorubicin group was significantly higher compared to the control group. (4.56 ± 1.19 vs. 2.03 ± 0.39; *p* = 0.036) (Figure 4).

### 3.2. Immunohistochemistry

**Figure 5:**
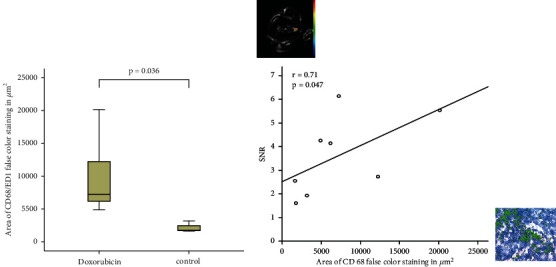
(a) Statistical analysis of macrophage CD68/ED1 staining using boxplot graphs shows the statistically significant enhancement in the doxorubicin group. (b) For the eight animals in total in the doxorubicin and control groups the plot shows the correlation between the SNR of fluorine enhancement in the myocardium (MRI, a picture of the ^19^F/^1^H overlay is shown on top of the *y*-axis) and the strength of the macrophage infiltration (CD68/ED1 histological staining, a picture is presented at the end of the abscissa displaying the areas above the color threshold in green) (significance level *p* < 0.05).

Immunohistochemical staining with a CD68/ED1 antibody demonstrated that the myocardium from doxorubicin-treated animals suffered from significantly stronger inflammation (10319 ± 6243 *μ*m^2^ vs. 2193 ± 849 *μ*m^2^; *p* = 0.036) (Figure 5(a)). The Spearman rank correlation showed a significantly strong correlation (*r* = 0.71 and *p* = 0.047) (Figure 5(b)).

## 4. Discussion

To our knowledge, this is the first *in vivo* study evaluating the application of a fluorine contrast agent in a rat myocarditis model using MRI systems at a clinically relevant magnetic field strength of 1.5 Tesla.

The strength of the ^19^F signal in the region of the myocardium was compared to the presence of infiltrated immune system cells in the myocardium as assessed by histological staining. The histological findings confirmed a significantly higher macrophage infiltration in the doxorubicin group. As macrophages were shown to be the primary sources of the active transport of fluorine nanoparticles to the inflammation site [9], our immunohistological analysis focused on detecting the relevant CD68/ED1-positive macrophages. However, other cell types are also suspected to interact with nanoparticles by phagocytosis, and they could therefore also have contributed to the detected signal [22].

MRI fluorine imaging relied on a particular pulse sequence. The adoption of the UTE-bSSFP sequence had been motivated by our expectations of a better detection of the ^19^F signal, but it was also beneficial for the preservation of the animals through a significant reduction of the total scan and anaesthesia time. To further reduce scanning time and stress on the animal, we had also chosen to renounce ECG-triggering, which most probably affected the imaging quality and deteriorated the SNR, particularly in the region of the moving myocardium.

Yet, performing the detection of fluorine signals at clinical field strengths means starting with a given handicap compared to other studies of experimental heart disease models. For instance, the works of van Heeswijk et al. and Jacoby et al. showed the potential of PFC-containing contrast agents in an experimental autoimmune and viral myocarditis model using a 9.4 Tesla MRI scanner [13, 14]. In order to reclaim sensitivity, our acquisitions were performed using a comparatively bigger isotropic voxel of 3 mm edge length (reconstructed to 1.5 mm). We consider that this was a viable trade-off for our acquisition strategy. First, if we posit that a coarser pixel might make up for the blurring effect of the motion of the myocardium, image acquisition triggering might not be essential (it was not used in our case). As mentioned before, this allowed for a shorter measurement duration. What is more, the larger rat heart volume offers an advantage compared to the afore mentioned studies relying on mouse models; the coarser resolution makes anatomical identification only slightly more difficult, and the minute anatomy of rats is rendered with sufficient sharpness. The same applies to ^19^F signals; a satisfactory analysis of the enrichment foci could be achieved. Finally, our proof-of-concept used a voxel size which would be considered rather fine in the context of human imaging. This brings us one step closer to the clinical setting, and we hypothesize that a marginally coarser resolution could still be necessary to make up for the possible lower efficiency of the clinical-grade spools. Solenoid coils are characterized by a high level of signal homogeneity and a high SNR in the entire coil volume. The phased array coils used in humans, on the other hand, have an inhomogeneous sensitivity profile, i.e., the SNR decreases with distance from the coil surface.

We should note that the concept of ultrashort TE sequence will probably receive further attention in the next years. Common blur-reducing acquisition techniques like triggering or motion compensation were not compatible with the implementation of UTE-bSSFP that we used; future refined versions might not only allow for a higher SNR or a narrower point-spread-function in the reconstruction algorithm, but also for access to traditional cardiac MRI techniques like retrospective imaging. This could be leveraged to explore the potential of ^19^F imaging in, among others, transient phenomena (angiography) or longitudinal studies (monitoring of the inflammation process).

In our sense, these results are an incentive to pursue preclinical studies with perfluorocarbon nanoemulsions on standard MRI clinical scanners, i.e., beyond the use of high-field equipment. Coincidentally, the protocols developed for our clinical system are directly “translatable” to bigger animal models.

As a final remark, the only PFC in the nanoemulsion used in our protocol is PFOB, which is widely recognized as safe and biologically inert. PFOB has been previously used extensively in human studies [23]. While the LD50 of a 25% (*w*/*v*) PFOB nanoemulsion was shown to be 30 g/kg in rats [24] our study used the much lower concentration of 2.1 g/kg in a 20% (*w*/*v*) PFOB nanoemulsion. In another study, perfluorocarbon concentrations of 1.35 g/kg to 1.80 g/kg were tested in humans without major complications [23, 25]. We could hypothesize that the *w*/*w* concentration of the PFOB-based contrast agent used in our study would have been safe if injected into a human being.

### 4.1. Study Limitation

Our study adopted a “proof of concept” approach and is limited by the group size. Due to the lack of ECG-triggered MRI acquisition, we also could not perform any kind of functional monitoring on the myocardium of the animals (e.g., contractility). Finally, the histological findings may underestimate the extent of inflammation caused by doxorubicin treatment due to the reduced resolution in the immunohistological evaluation.

## 5. Conclusions

We demonstrated the successful implementation of a PFOB nanoemulsion in the diagnostics of experimental myocarditis using the clinically established field strength of 1.5 Tesla. In a more general stance, this study contributes to the evidence that perfluorocarbon nanoemulsions can become a prolific tool for inflammation imaging and is thus a promising element for translational medicine.

## Data Availability

The data regarding to this publication is available from the corresponding author upon request.
